# Same same but different? How blood and lymphatic vessels induce cell contact inhibition

**DOI:** 10.1042/BST20240573

**Published:** 2025-02-06

**Authors:** Claudia Carlantoni, Leon M.H. Liekfeld, Manu Beerens, Maike Frye

**Affiliations:** 1 Institute of Clinical Chemistry and Laboratory Medicine, University Medical Center Hamburg-Eppendorf, Hamburg 20246, Germany; 2 German Centre of Cardiovascular Research (DZHK), Partner Site Hamburg, Luebeck, Kiel, Hamburg, Germany

**Keywords:** (lymph)angiogenesis, blood endothelial cells, cadherins, cell cycle, CIP, contact inhibition, contact inhibition of proliferation, Hippo pathway, lymphatic endothelial cells, Notch, PDE2A, proliferation, vascular development

## Abstract

Endothelial cells (ECs) migrate, sprout, and proliferate in response to (lymph)angiogenic mitogens, such as vascular endothelial growth factors. When ECs reach high confluency and encounter spatial confinement, they establish mature cell–cell junctions, reduce proliferation, and enter a quiescent state through a process known as contact inhibition. However, EC quiescence is modulated not only by spatial confinement but also by other mechano-environmental factors, including blood or lymph flow and extracellular matrix properties. Changes in physical forces and intracellular signaling can disrupt contact inhibition, resulting in aberrant proliferation and vascular dysfunction. Therefore, it is critical to understand the mechanisms by which endothelial cells regulate contact inhibition. While contact inhibition has been well studied in blood endothelial cells (BECs), its regulation in lymphatic endothelial cells (LECs) remains largely unexplored. Here, we review the current knowledge on extrinsic stimuli and intrinsic molecular pathways that govern endothelial contact inhibition and highlight nuanced differences between BECs and LECs. Furthermore, we provide perspectives for future research on lymphatic contact inhibition. A deeper understanding of the BEC- and LEC-specific pathways underlying contact inhibition may enable targeted modulation of this process in blood or lymphatic vessels with relevance to lymphatic or blood vascular-specific disorders.

## Introduction

The term ‘contact inhibition’ was first introduced in the 1950s when Abercrombie and Heaysman observed that fibroblast-like cells derived from chick heart explants altered their direction of movement to avoid colliding with other cells in a two-dimensional (2D) culture [1]. This phenomenon, referred to as contact inhibition of locomotion, facilitated the maintenance of a cell monolayer architecture by preventing cells from overgrowing one another [1]. A decade later, the term ‘contact inhibition’ was also adopted to describe the cell cycle arrest observed for many cell types in culture, a process termed contact inhibition of proliferation [2]. Although initially studied in *in vitro* cell cultures of fibroblasts [3], epithelial cells [4–6], endothelial cells (ECs) [7–9], or cardiomyocytes [10], contact inhibition has been demonstrated to be a fundamental mechanism in tissue growth and development [11–14], as well as wound healing and homeostasis [15,16]. As early as 1967, it was demonstrated that melanoma cells maintained exponential colony growth rates, whereas fibroblasts shifted to a linear growth phase as cell density increased [17]. Concordantly, the loss of contact inhibition was recognized as a hallmark of cancer cells in tumors [18].

### Cell cycle regulation

Contact inhibition is intrinsically linked to cell cycle regulation, as increased cell density induces arrest in the gap 0 (G0)/gap 1 (G1) phase, effectively preventing further progression through the cell cycle. The cell cycle is historically divided into G1, DNA synthesis (S), gap 2 (G2) – together forming the interphase – and mitosis (M) phases. Cell cycle transitions from G1 to S phase, as well as from G2 to M phase, are tightly controlled by cyclins and cyclin-dependent kinase (CDK) complexes [19]. Specifically, cyclin D-CDK4/CDK6 regulates G1 phase progression, the cyclin E-CDK2 complex modulates the transition from G1 to S phase, cyclin A-CDK2 are involved in S phase, cyclin A-CDK1 regulate G2 phase, and, lastly, cyclin B-CDK1 complex is involved in G2 to M transition [19,20]. Moreover, the transition to the S phase requires further inactivation of the Retinoblastoma (Rb) protein through its phosphorylation by the cyclin D-CDK4/CDK6 complex. This phosphorylation event releases the E2F-DP (dimerization partner) complex, enabling the subsequent expression of cyclins E and A and the progression through the cell cycle [21,22].

Each phase of the cell cycle can be inhibited by cyclin-dependent kinase inhibitors (CDKIs), which are categorized into two families: the Cip/Kip family (or CDKN1 family), comprising p21 (human gene name: *CDKN1A*) [23], p27 (*CDKN1B*), and p57 (*CDKN1C*) [24] and the INK4 family [25], consisting of p16 and p14ARF (or p19ARF in mice, both encoded by *CDKN2A* and *Cdkn2,* respectively), as well as p15 (*CDKN2B*), p18 (*CDKN2C*), and p19 (*CDKN2D*). The two families of CDKIs encode distinct proteins with different biochemical and functional characteristics, reflecting their specific roles in cell cycle regulation [26,27]. The Cip/Kip family primarily inhibits CDK1 and CDK2, effectively regulating the cell cycle at various checkpoints [28]. In contrast, the members of the INK4 family predominantly target CDK4 and CDK6, playing a crucial role in controlling the transition from G1 to S phase. Interestingly, global knockout mice for the majority of the *Cdkn1* and *Cdkn2* genes do not show major developmental or proliferation defects, likely due to genetic redundancy or compensation [29]. Differential organ-specific expression profiles of CDKIs and various post-translational modifications [30,31] further influence their function independently of their transcriptional dynamics.

In addition to the four canonical phases of the cell cycle, cells can also enter the G0 phase. During this phase, inhibitors from the CDKN2 family are typically up-regulated, and transcriptional repression of cell cycle activators occurs, resulting in a state of quiescence and cell cycle arrest[32]. Upon removal of the stimuli that induce cell cycle arrest, cells can exit their quiescent state and reactivate the cell cycle. However, the time required to re-enter the S phase depends on the ‘depth of quiescence’ experienced; the longer the cells remain quiescent, the more time they require to initiate the S phase [33,34]. This relationship highlights the dynamic nature of cellular responses to prolonged inactivity and their capacity for re-entry into active proliferation. It is important to note that quiescence is not synonymous with senescence. In contrast with quiescent cells that retain their potential for reactivation, senescent cells are incapable of responding to mitogenic stimuli and, as a result, cannot re-enter the cell cycle [35].

### The concept of contact inhibition

Contact inhibition is established in stages, beginning with an initial increase in cell size that triggers entry into the S phase, leading to subsequent cell proliferation [36]. This increase in cell size must reach a substrate-dependent threshold, highlighting a crucial role of the mechanical microenvironment in the regulation of contact inhibition [36,37]. Substrate stiffening was also shown to sensitize epithelial cells to epidermal growth factor (EGF) as moderate stiffening of the matrix reduced the threshold amount of EGF needed to over-ride contact inhibition by over 100-fold 38.

Subsequently, in a confluent monolayer, contact inhibition occurs following the suppression of cell motility and is triggered when mechanical constraints on local expansion cause cell divisions to reduce individual cell area [39]. Indeed, the mechanical pressure of confinement also plays a crucial role in establishing long-term contact inhibition. This process involves the translocation of β-catenin from cell junctions to the nucleus, indicating that β-catenin mediates the establishment of a pressure threshold that triggers contact inhibition [40].

However, even in confluent monolayer, regions with higher proliferation rates can be identified in both epithelial and EC cultures [41]. This has been correlated with mechanical stimulation caused by the geometry of the environment, such as the corners and edges of cell culture dishes [41]. Interestingly, not only do cells that directly experience mechanical stimuli, such as available space, re-enter the cell cycle, but also cells located farther away from these stimuli also begin to proliferate, indicating that these mechanical cues can be sensed or transmitted over a certain distance from the source [41].

These concepts have been reinforced recently through **computational models** where tissue growth varies depending on the cell sensitivity to contact inhibition (i.e. intrinsic response to growth factors) and the elastic modulus of the substrate (stiffness) [42]. Mechanical feedback between tissue confinement and individual cell growth enhances cell proliferation at tissue boundaries, while growth in the center mass is suppressed, reflecting experimental observations in epithelial cells previously described [36,41]. Notably, adjusting the model to higher elasticity (stiffness) led to increased bulk growth, in line with the previous notion that many cell types exhibit greater proliferative capacity when exposed to stiffer substrates [43]. Intermediate levels of both contact inhibition and stiffness replicate the growth patterns observed in tissues during development and homeostasis, suggesting a complex interplay between microenvironmental and intrinsic cues [42]. Other modeling approaches demonstrate that, before cells encounter constraints or confinement and when contact inhibition is minimal, cell colonies grow exponentially over time, driven solely by the proliferation rate [44]. Later, the colony boundary moves at a constant speed, determined exclusively by the migration rate of individual cells and independent of the proliferation rate [44].

Moreover, significant differences in cell velocity, age, and stress distributions were observed between non-migrating and migrating cells, as analyzed using a multiphase field model [45]. Importantly, numerical simulations show that, consistent with experimental findings, the loss of contact inhibition is a sufficient mechanism to explain the increase in the proportion of tumor cells [46], demonstrating that the molecular mechanisms that define contact inhibition may constitute druggable targets. Computational models primarily focused on angiogenic processes at the tissue level [47] could potentially be expanded in the future to include modeling of contact inhibition.

### Adhesion molecules and the Hippo pathway: key regulators of contact inhibition

Many studies highlight the importance of cell adhesion molecules in the establishment of contact inhibition. For example, in epithelial carcinoma cells, E-cadherin controls an increase in expression of the CDKN1 protein p27 to trigger cell cycle arrest [4]. Notably, nonadherent mouse mammary carcinoma cells transfected with E-cadherin showed increased adhesion within multicellular spheroids and reduced proliferation [4]. In contrast, in HCT116 human colon carcinoma cell line, the CDKI p21 was shown to reduce E-cadherin expression, which was necessary to form multicellular spheroids that can proliferate [48]. Lack of functional p21 and E-cadherin caused proliferation stop and apoptosis [48].

In another model using the SW480 colorectal tumor cell line, it was, however, shown that cells lacking E-cadherin expression rather increased their proliferative potential through the activation of the β-catenin/TCF pathway, demonstrating that not only the actual adhesive function of junctional proteins but also cytoplasmic effectors of cell–cell contacts are involved in the regulation of contact inhibition [49].

Although growth factors are among the major players inducing cell proliferation (reviewed in [50–53]), the cellular response to them may vary depending also on the state of cell–cell adhesion. For example, modulating cell–cell contacts through different substrates or E-cadherin overexpression can induce contact inhibition even at higher EGF concentrations typically permissive for proliferation. This suggests that the threshold required to activate growth factor-mediated cell cycle activity is adjustable and depends on the local balance between growth factor levels and cell–cell contact states [54].

E-cadherin and its associated β-catenin are also responsive to mechanical stress in epithelial cells, with static biaxial stretch leading to nuclear translocation of YAP1 (effector of the Hippo pathway) and β-catenin [55]. While YAP1 remains in the nucleus for only a few hours, β-catenin localizes in the nucleus for up to 24-hour post-stimulus. This suggests that the Hippo pathway is more important in sensing local and transient changes in the mechanical microenvironment and cell–cell contacts and leads to a more rapid response [40,55]. Similarly, high cellular density in epithelial cells or a soft extracellular matrix (ECM) activates the Hippo pathway, increases LATS1/2 kinase activity, and subsequently inactivates YAP1/TAZ [6]. The inactivation or cytosolic translocation of YAP1/TAZ reduces the expression of myosin-II genes and leads to the loss of actin stress fibers, which, in turn, impairs autophagosome activity and reduces proliferation [6]. In line with this, the F-actin-capping/severing proteins Cofilin, CapZ, and Gelsolin have been identified as essential gatekeepers that limit YAP/TAZ activity in epithelial cells experiencing low mechanical stresses during contact inhibition [56]. Furthermore, more recent studies have shown that the activation of YAP1 through the annexin A2 inhibitor PY-60 leads to escape from contact inhibition, allowing keratinocytes to continue proliferating even at high cell density [57].

## Contact inhibition in blood endothelial cells

Blood endothelial cells (BECs) are sensitive to contact inhibition both *in vitro* and *in vivo*, where BECs undergo low turnover in large, as well as small, caliber vessels [58,59]. The homeostatic, quiescent state of BECs can be considered that of a confluent monolayer, as mature vessels are lined with an EC monolayer naturally attached in a 2D manner to the underlying ECM [43]. While the exact cell cycle arrest states of different BEC subtypes have not been comprehensively mapped, human umbilical vein ECs (HUVECs), bovine pulmonary arterial ECs, and human corneal ECs have been described to arrest in the G1 phase [60,61]. However, in case of vessel injuries, tumor angiogenesis [62], and in vascular diseases [63], BECs are reactivated to induce proliferation and migration. The shift between the quiescent, contact-inhibited state and the proliferative state in BECs is also driven by growth factors and junctional rearrangements, which activate or repress intracellular pathways [64].

### Mitogenic control of blood endothelial cell contact inhibition

For example, the binding of the major angiogenic mitogen vascular endothelial growth factor (VEGF) to VEGF receptor 2 (VEGFR2) activates and internalizes the receptor, triggering EC proliferation to support the expansion of new and tumor-associated blood vessels (reviewed in [65]). Other mitogenic stimuli, such as fibroblast growth factor (FGF) or fetal calf serum, or low-density culture conditions, activate p42/p44 mitogen-activated protein kinase (MAPK), also known as ERK1/2, in BECs and allow them to enter the cell cycle [66], in a manner similarly described for epithelial cells [67] and fibroblasts [68]. Notably, this process can be reversed by the depletion of mitogenic signals and/or an increase in monolayer confluency, demonstrating a finely tuned regulatory mechanism for cell cycle arrest of the blood endothelium [66]. Interestingly, recent work by Pontes-Quero et al*.* challenges the traditional assumption that increased growth factor concentration, as well as the resulting mitogenic activity, drives both endothelial proliferation and sprouting [69]. Instead, very high mitogenic stimulation induced by VEGF or Notch inhibition actually arrested the proliferation of angiogenic tip cells in the retina [69]. The study identified a bell-shaped dose–response to VEGF and MAPK activity, regulated by Notch and p21, which determines whether BECs sprout/migrate, proliferate, or enter a quiescent state [69]. If and how the adhesive properties of BECs co-determine these cellular responses remains to be explored.

In contact-inhibited BECs, the activity of phosphatases is increased leading to down-regulation of ERK and phosphatidylinositol 3-kinase (PI3 K)/Akt signaling [70]. The process is reversible, as demonstrated by the use of phosphatase inhibitors, which induce cell cycle progression [70].

### Junctional control of blood endothelial cell contact inhibition

Vascular endothelial (VE)-cadherin is the major adhesion molecule in blood endothelial cell–cell contacts [71–73]. Similar to epithelial E-cadherin, the absence of VE-cadherin in BECs results in continuous proliferation [7]. Vice versa, high expression of VE-cadherin was associated with reduced activity of VEGFR2 and ERK signaling, as well as increased activity of the high cell density–enhanced protein tyrosine phosphatase 1 (DEP-1)/CD148 [7]. Furthermore, β-catenin-mediated association of VE-cadherin and VEGFR2 was necessary to induce cell cycle arrest [7] and VE-cadherin retained VEGFR2 at the membrane, thereby preventing its internalization into signaling compartments [74]. In contrast with low ERK activity triggered by contact inhibition, shear stress-induced up-regulation of connexin 37 in BECs requires ERK phosphorylation to increase p27 expression and promote G1 arrest [75], indicating that absolute levels of ERK and its activity are not the sole determinators of BEC proliferation.

Another mechanism by which VE-cadherin regulates the cell cycle and contact inhibition in BECs is through phosphorylation of its tyrosine 685 residue and its subsequent interaction with the protein tyrosine kinase C-terminal Src kinase (Csk) [76]. In correlation with cell monolayer density, Csk then transduces the signal of tight cell–cell contact formation intracellularly to induce contact inhibition [76]. While not directly involved in BEC contact inhibition, VE-cadherin-mediated up-regulation of Claudin 5 (CLDN5) is necessary for forming tight junctions in BECs, as demonstrated in *in vitro* and *ex vivo* allantois explants. However, during blood vessel homeostasis, CLDN5 is differentially expressed in arteries versus veins [77] and does not control HUVEC barrier properties [78], suggesting its potential vascular bed-specific role in contact inhibition.

Other junctional regulators have been shown to be differentially expressed in sub-confluent versus confluent BEC cultures and, therefore, may play a role in the establishment of contact inhibition. N-cadherin is present at the junctions of sub-confluent BECs, where it is involved in forming the first cell–cell connections, but N-cadherin is excluded from stable cell–cell junctions with increasing monolayer density [79]. ICAM-2 expression, although already present in sub-confluent BECs, was found to control N-cadherin and VE-cadherin recruitment into endothelial junctions through recruitment and activity of ezrin, radixin, and moesin (ERM) and Rac1 proteins to induce contact inhibition [79]. Notably, *in vivo* deletion of VE-cadherin did not result in N-cadherin localization to junctions [71], suggesting a more complex regulation of the cadherins *in vivo* with potential consequences for cell cycle states.

### Mechanical control of blood endothelial cell contact inhibition

BECs constantly interact with mechanical forces at their luminal side, such as flow-induced shear stress, and on their abluminal side via changes in stiffness and stretch [43]. Recent work has highlighted molecular differences in laminar flow-induced quiescence among BEC types. Arterial ECs exposed to flow initially entered deep quiescence and then transitioned to a shallow homeostatic quiescence, while venous ECs maintained a stable deep quiescence [80]. p27 was essential for flow-mediated quiescence, with expression levels correlating with quiescence depth. Mechanistically, the Notch and bone morphogenetic protein (BMP) target HES1 and ID3, respectively, and act as p27 repressors, adjusting quiescence depth by lowering p27 levels to promote shallow quiescence [80].

In ECs, proliferation decreases in soft 2D substrates and increases in stiff 2D substrates [43]. For example, HUVECs cultured on more compliant matrices (1 kilopascal, kPa) showed increased expression and clustering of VEGFR2, while stiffer matrices (10 kPa) induced increased VEGFR2 internalization and signaling [81]. This switch was mediated by Rho activity and actin contractility [81]. In a confluent endothelial monolayer, however, stiffness-enhanced VEGF signaling is no longer observed, suggesting that this mechanism is specific to actively proliferating cells and angiogenic processes and suppressed once contact inhibition is established [81]. Notably, not only ECM stiffness but also the ECM’s capacity to retain mitogens might play a role in BEC contact inhibition [82]. Furthermore, mechanosensitive YAP1 signaling has been shown to regulate endothelial contact inhibition, as the loss of the YAP1 regulator DLC1 (deleted in liver cancer 1) leads to a loss of contact inhibition, while YAP silencing prevents this effect [83].

### Notch signaling as a central regulator of contact inhibition in blood endothelial cells

Notch signaling has been implicated in cell fate control during development, where it requires cell–cell contact to activate lateral inhibition [84]. Since the early 2000s, several studies have demonstrated how Notch employs a similar mechanism to induce contact inhibition in BECs. When BECs are plated at low, medium, or high density, downstream Notch genes are up-regulated in correlation with increasing cell confluency [8,85]. An increase in Notch activation correlates with a reduction in p21 and an increase in p27 expression, indicating that Notch plays a key role in establishing BEC quiescence and contact inhibition [8,85]. Additionally, Notch signaling controlled BEC contact inhibition through the regulation of the minichromosome maintenance (MCM) proteins 2 and 6 [86]. In this case, Notch activation down-regulates MCM2 and MCM6 expressions, which, in turn, reduces Rb phosphorylation, thereby blocking cell cycle progression [86].

While *in vitro* studies have shown that active Notch signaling consistently acts as a contact inhibition signal, *in vivo* models of retinal angiogenesis reveal that its role is highly context-dependent [69] discussed above. At very low VEGF signaling levels, retinal ECs remain quiescent with active Notch signaling that suppresses ERK activity and cell proliferation. Stalk cells operate under balanced Notch and VEGF signaling, producing an ERK activity level optimal for controlled proliferation. In contrast, tip cells experience high VEGF and low Notch signaling, leading to elevated ERK activity, which induces p21, cell cycle arrest, and promotes cell sprouting and migration [69].

Notch signaling has also been implicated in arterialization, where it is coupled with the suppression of the BEC cell cycle. Using inducible genetic mosaics, it was shown that although BECs with high VEGF and Notch signaling are preferentially located in arterial vascular beds, Notch does not directly activate an arterial genetic program but instead suppresses MYC-driven metabolic and cell cycle activities [87]. Consistent with this, microRNA-218 (miR-218) has been identified as a downstream effector of active Notch signaling in quiescent BECs [88]. Induction of miR-218 expression attenuates MYC activity, thereby repressing BEC proliferation and promoting contact inhibition [88].

## Contact inhibition in lymphatic endothelial cells

**Figure 1:**
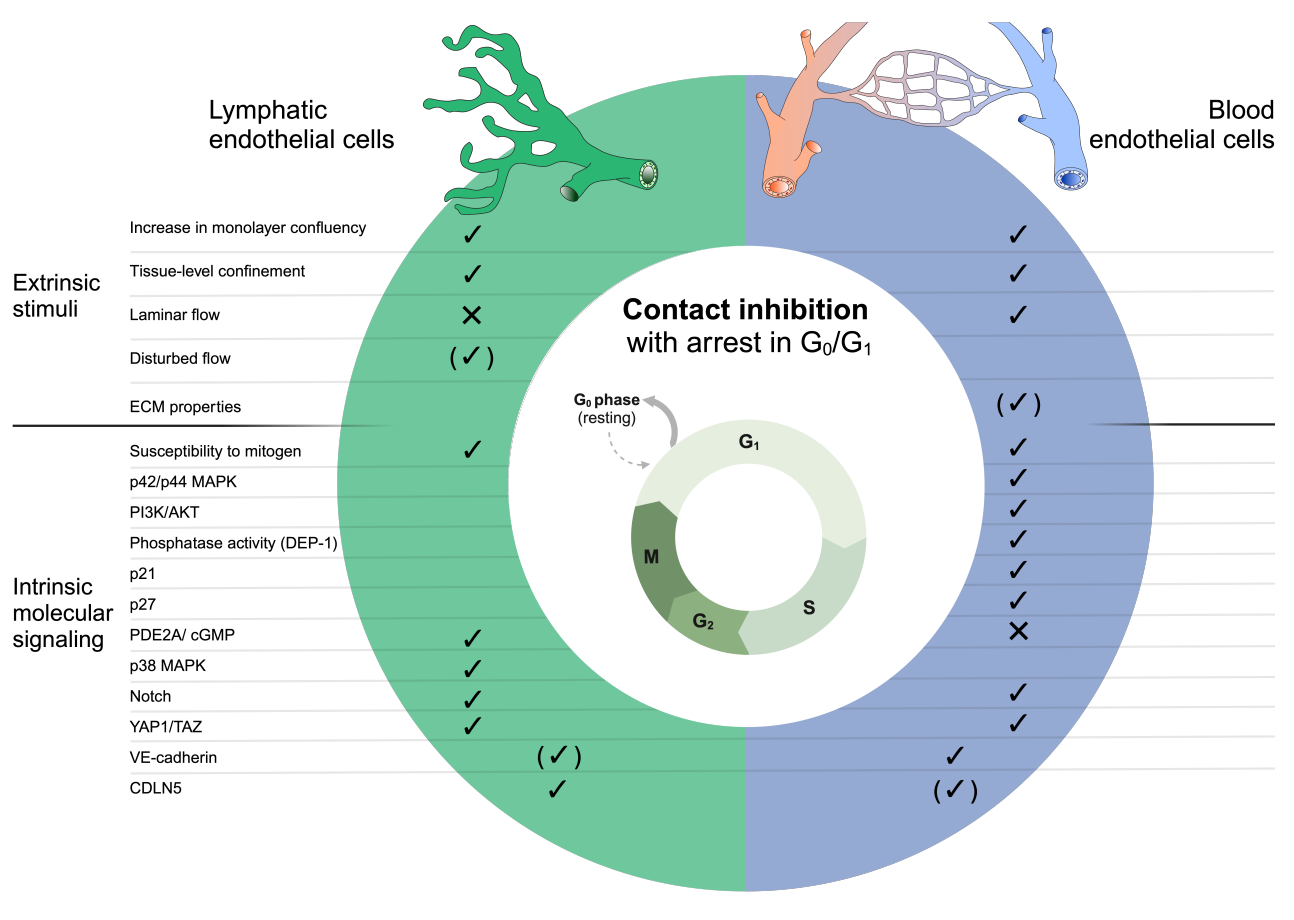
Mechanisms of contact inhibition in lymphatic and blood endothelial cells. Blank spaces indicate that the extrinsic stimulus or molecular pathway has not been studied in the context of lymphatic or blood endothelial contact inhibition. (✓) denotes context-specific regulation of contact inhibition. Notably, several key regulators of contact inhibition have not yet been specifically studied in lymphatic endothelial cells.

Although contact inhibition has been extensively studied in BECs, its regulation in lymphatic endothelial cells (LECs) remains largely unknown (Figure 1). The authors and others have shown that the regulation of endothelial barrier function employs different molecules in BECs and LECs, even when expression patterns and levels appear similar across lymphatic and blood vascular beds and organs. This applies to molecules such as VE-cadherin [71,89] and CLDN5 [77,90], as well as EphB4 [90,91], and suggests that certain aspects of contact inhibition in LECs may also differ from those in BECs.

### The PDE2A/cGMP/p38/MAPK/Notch axis controls lymphatic endothelial cell contact inhibition

Phosphodiesterase 2A (PDE2A) is a phosphodiesterase with dual specificity, which hydrolyzes cyclic adenosine monophosphate (cAMP) to AMP and cyclic guanosine monophosphate (cGMP) to GMP [92]. The loss of PDE2A in BECs was correlated with increased cAMP levels and dysregulated blood vessel barriers during homeostasis and inflammation [93,94]. However, despite these roles, PDE2A is not essential for proper blood vessel formation *in vitro* and *in vivo* [14].

Recently, we have shown that LECs induce contact inhibition through a previously unappreciated PDE2A-controlled cGMP/p38 MAPK/Notch axis [14]. We demonstrated that the loss of lymphatic PDE2A causes embryonic lymphatic dysplasia *in vivo*, as well as increased cGMP levels, defective junctions, and down-regulation of the major lymphatic junctional regulator CLDN5 *in vitro* [14]. Interestingly, VE-cadherin expression was not significantly altered in the absence of PDE2A [14].

An RNA sequencing approach comparing low-confluency (CLDN5^Low^) and high-confluency (CLDN5^High^) LECs revealed both similar and distinct gene expression changes in junctional and contact inhibition/proliferation genes, compared with BECs. Notably, *FLT4* (the coding gene for VEGFR3) expression was significantly elevated in contact-inhibited LECs but not BECs [14], potentially suggesting the distinct responsiveness to VEGFC compared with VEGF/VEGFR2 in LECs versus BECs, respectively.

Using the same rationale but comparing CLDN5^high^ LECs in the presence and absence of PDE2A reversed gene expression, with proliferation genes being up-regulated and junctional genes being down-regulated in the absence of PDE2A. In the absence of *Pde2a* in lymphatic vessels, LECs showed moderate but continuous proliferation in mouse embryonic back skins which coincided with compromised LEC contact inhibition [14]. The moderate, rather than hyperproliferative, growth is likely due to a decline in mitogenic signaling at later stages of lymphatic development.

The lymphatic defects observed upon PDE2A loss were attributed to the enzyme’s unique function in LECs, where it selectively hydrolyzes cGMP over cAMP. The resulting elevation in cGMP levels in LECs disrupts junctional integrity, leading to the loss of contact inhibition. Elevated cGMP levels also led to increased p38 phosphorylation, a pathway previously implicated in contact inhibition of other cell types [95,96] but not in BECs. Downstream of p38, LECs activated Notch signaling to promote contact inhibition [14], aligning with the previously established role of Notch in BEC contact inhibition.

### Mechanical control of lymphatic endothelial cell contact inhibition

Unlike BECs [80], LECs do not initiate contact inhibition and quiescence when exposed to steady laminar flow [97,98]. For example, in response to laminar flow, LECs, but not BECs, showed increased proliferation through regulation of VEGFA, VEGFC, FGFR3, and p53/CDKN1C. ORAI1, a subunit of the calcium release-activated calcium channel, was identified as an early mediator of these shear stress responses and proliferation in LECs [97]. Moreover, flow-mediated proliferation was accompanied by the loss of lymphatic contact inhibition during homeostasis in mice, indicating that LECs possess unique regulatory mechanisms to finetune contact inhibition and proliferation.

Furthermore, these mechanisms display heterogeneity throughout the lymphatic vascular tree. Flow also controlled the loss of lymphatic contact inhibition during homeostasis in mice. Adult LEC turnover and proliferation was shown to be confined to valve regions of collecting vessels, with valve cells displaying the shortest lifespan [99]. Exposure to low recirculating flow, modeled by oscillatory shear stress (OSS) *in vitro*, induced valve cell proliferation via mTORC1 signaling to support the renewal of valve LECs, which are naturally subjected to higher mechanical stress [99]. In contrast, high recirculating, disturbed flow in lymphatic vessels (and high OSS *in vitro*) cooperates with the transcription factor FOXC2 to induce quiescence and ensure lifelong stability of the lymphatic vasculature. The loss of FOXC2 conferred abnormal shear stress sensing, activated YAP1/TAZ signaling, and promoted junction disassembly and entry into the cell cycle *in vitro* [98].

Additionally, both fluid accumulation (by increasing the amount of interstitial fluid in mouse embryos in *‘gain-of-fluid*’ experiments) and resulting LEC stretching were shown to induce VEGFR3/β1-integrin-mediated proliferation [100]. Conversely, *‘loss-of-fluid*’ experiments revealed reduced LEC proliferation [100], suggesting that this mechanism could also play a role for LEC contact inhibition at a later stage.

Finally, and importantly, studies in zebrafish have shown that precise regulation of the CDKIs p27 and p21 is essential to control lymphatic sprouting [101,102]. Whether these underlying mechanisms also contribute to lymphatic contact inhibition later in development remains to be determined.

## Conclusion

Stable and quiescent endothelial cell junctions are of vital importance to guarantee blood and lymph flow without leakage. Endothelial contact inhibition controls quiescence and prevents uncontrolled proliferation, a condition associated with various vascular diseases, such as hemangiomas, vascular malformations, atherosclerosis, and psoriasis, as well as tumor (lymph)angiogenesis. Blood endothelial contact inhibition has been extensively studied, showing that many of its key regulators are shared across cell types, including fibroblasts and epithelial cells. In contrast, mechanisms of lymphatic contact inhibition are still understudied. Interestingly, while Notch activation plays a similar role in promoting contact inhibition in both BECs and LECs, our findings and those of others indicate that certain extrinsic factors, such as differential types of fluid flow, and molecular pathways, like the PDE2A/cGMP/p38 MAPK axis, are specific to BECs or LECs (Figure 1). EC-specific control of contact inhibition could provide the opportunity to modulate contact inhibition in selected vessel types.

**Perspectives**

- In high-confluency monolayers and under spatial confinement, endothelial cells form mature cell–cell junctions and reduce proliferation. This process, referred to as contact inhibition, controls endothelial quiescence and prevents uncontrolled proliferation, a condition associated with various vascular diseases.
- Blood endothelial contact inhibition has been extensively studied showing that many of its key regulators, such as PI3K/AKT, Notch, Yap, and cadherins, are shared across cell types, including fibroblasts and epithelial cells. Furthermore, many additional signaling pathways have been implicated in regulating endothelial proliferation and the formation of endothelial contacts. However, whether these pathways are also linked to adhesive processes during contact inhibition remains to be experimentally determined.
- The regulation of contact inhibition in lymphatic endothelial cells is, however, still understudied. A select few studies show that while LECs and BECs share common mechanisms of contact inhibition, nuanced differences are now being identified, which could allow for targeted modulation of blood versus lymphatic vessels.

## Abbreviations

BECs: Blood Endothelial Cells
BMP: bone morphogenetic protein
CDK: Cyclin-Dependent Kinases
CDKIs: Cyclin-Dependent Kinase Inhibitors
ECM: Extracellular Matrix
EGF: Epidermal Growth Factor
ERM: Ezrin, Radixin, and Moesin
FGF: Fibroblast Growth Factor
HUVECs: Human Umbilical Vein Endothelial Cells
LECs: Lymphatic Endothelial Cells
MAPK: Mitogen-Activated Protein Kinase
MCM: Minichromosome Maintenance
Rb: Retinoblastoma Protein
VE: Vascular Endothelial
VEGFs: Vascular Endothelial Growth Factors
cAMP: Cyclic Adenosine Monophosphate
cGMP: Cyclic Guanosine Monophosphate
